# Unusual isothermal multimerization and amplification by the strand-displacing DNA polymerases with reverse transcription activities

**DOI:** 10.1038/s41598-017-13324-0

**Published:** 2017-10-24

**Authors:** Guoping Wang, Xiong Ding, Jiumei Hu, Wenshuai Wu, Jingjing Sun, Ying Mu

**Affiliations:** 1 Research Center for Analytical Instrumentation, Institute of Cyber-Systems and Control, State Key Laboratory of Industrial Control Technology, Zhejiang University, Hangzhou, 310058 P. R. China; 2 College of Life Sciences, Zhejiang University, Hangzhou, 310058 P. R. China

## Abstract

Existing isothermal nucleic acid amplification (INAA) relying on the strand displacement activity of DNA polymerase usually requires at least two primers. However, in this paper, we report an unusual isothermal multimerization and amplification (UIMA) which only needs one primer and is efficiently initiated by the strand-displacing DNA polymerases with reverse transcription activities. On electrophoresis, the products of UIMA present a cascade-shape band and they are confirmed to be multimeric DNAs with repeated target sequences. In contrast to current methods, UIMA is simple to product multimeric DNA, due to the independent of multiple primers and rolling circle structures. Through assaying the synthesized single-stranded DNA targets, UIMA performs high sensitivity and specificity, as well as the universality. In addition, a plausible mechanism of UIMA is proposed, involving short DNA bending, mismatch extension, and template slippage. UIMA is a good explanation for why nonspecific amplification easily happens in existing INAAs. As the simplest INAA till now, UIMA provides a new insight for deeply understanding INAA and opens a new avenue for thoroughly addressing nonspecific amplification.

## Introduction

As point-of-care testing (POCT) techniques for molecular diagnosis^1–4^, isothermal nucleic acid amplification (INAA) is advantageous over conventional PCR, due to its high specificity, efficiency, rapidity, and the independent of thermal cyclers^5–8^. The mechanism of existing INAAs is commonly based on the strand displacement activity of DNA polymerases and the self-initiated DNA synthesis of hybrid primer’s extension product, such as, loop-mediated amplification (LAMP)^9^, cross priming amplification (CPA)^10^, and isothermal multiple-self-matching-initiated amplification (IMSA)^11^. To achieve high sensitivity and specificity, these INAAs entail multiplex primers targeting multiple sequence sites, which inevitably enhances the probability of nonspecific amplification^12^. Even through electrophoresis analysis, nonspecific amplifications are indistinguishable, since their products are the same as those of specific amplifications^10,13^. Besides, it is not easy to design multiplex primers with high efficiency.

As alternatives to multiple primers-based INAAs, polymerase spiral reaction (PSR)^14^ and linear target isothermal multimerization amplification (LIMA)^15^ are preferable. They only require two primers and two target sites, lowering the difficult on primer design. On reaction principle, PSR employs two primers with non-template sequences at their 5′ ends to form a spiral structure based on which vortex-type amplification is self-initiated. Interestingly, in a further study of PSR, the primers turned to two pairs and recognized five target sites to improve the reaction velocity^16^. Compared to PSR, LIMA is supposed to be the real sense of isothermal PCR-like technique, because its two primers are entirely obtained from the target sequences^15^. Unlike PCR, the products of LIMA are larger than the amplified region and display a ladder-shape band on electrophoresis. To be developed as a POCT approach, however, LIMA compromises on robustness and reproducibility of detection^15^.

LIMA is strictly template-dependent and usually appears in some INAAs as nonspecific amplification, for instance, the loop primers-contained LAMP. In contrast to conventional LAMP, six primers-based version performs rapidness, owing to the addition of two loop primers^17^. As reported previously, the loop primers could open the double-stranded DNA (dsDNA) products with loop structures and thus accelerate the reaction^17^. Despite that explanation, the acceleration effect is likely attributed to LIMAs mingling in the LAMP, such as those initiated by the primer pair of F3 and LF or the B3 and LB. Even in conventional LAMP, by-products also emerge due to the multimerization of primers and target DNAs^18^. Although it is beneficial to expedite the main amplification in INAAs, LIMA as nonspecific amplification results in complicated amplicons and influences downstream analysis (e.g. enzyme digestion). A stark example is the single crossing CPA^10^. On mechanism, single crossing CPA should not produce DNA fragments with large sizes, but electrophoresis obviously indicates large by-products, leading to incomplete digestion of the products. Probably, these resulting by-products are from the LIMAs caused by CPA’s primers of 4 s and 3a/2a/5a.

In this study, we concentrate on LIMA and find that LIMA actually belongs to an unusual isothermal multimerization and amplification (UIMA) which only requires one primer. On assaying the synthesized single-stranded DNA (ssDNA) targets, UIMA performs high sensitivity and specificity, as well as the universality. Resembling LIMA, UIMA’s products are also multimeric DNAs with the sequences derived from the target^15^. To achieve rapid UIMA, the duplex formed when the primer recognizes the site should have an overhang at template’s 3′ end. After amplification, this overhang is entirely incorporated into the products. More importantly, UIMA is only efficiently initiated by the strand-displacing DNA polymerases with reverse transcription (RT) activities, such as *Bst* DNA polymerase, Bsm DNA polymerase, and BcaBEST DNA polymerase. UIMA also presents even using the reverse transcriptase of MHM, while it hardly happens utilizing the polymerases without RT activities. According to the results of sequencing analysis, a plausible mechanism of UIMA is proposed, which involves short DNA bending, mismatch extension, and template slippage. UIMA is a good explanation for why some INAAs always contain large size of DNA products which are not deduced from their schemes. When taking the ssDNA template as one primer, UIMA becomes primer dimers-based nonspecific amplification. Hence, our findings here also hint a new strategy to address nonspecific amplification thoroughly, namely removing RT activity from strand-displacing DNA polymerase, rather than using costly fluorescence probes^19–21^, new dyes^22–24^ and nano-technologies^25,26^.

## Results

### UIMA was a template-dependent amplification reaction

To investigate whether UIMA was a template-dependent reaction, a synthesized ssDNA with the primer-recognized site was used as the template and meanwhile three control tests of no-template control (NTC), no-primer control (NPC), and no-enzyme control (NEC) were set up. NTC, NPC, and NEC shared the same UIMA’s reaction system, except for their own absent component. The strand-displacing DNA polymerase used here was *Bst* WarmStart DNA polymerase (*Bst* WS).

**Figure 1:**
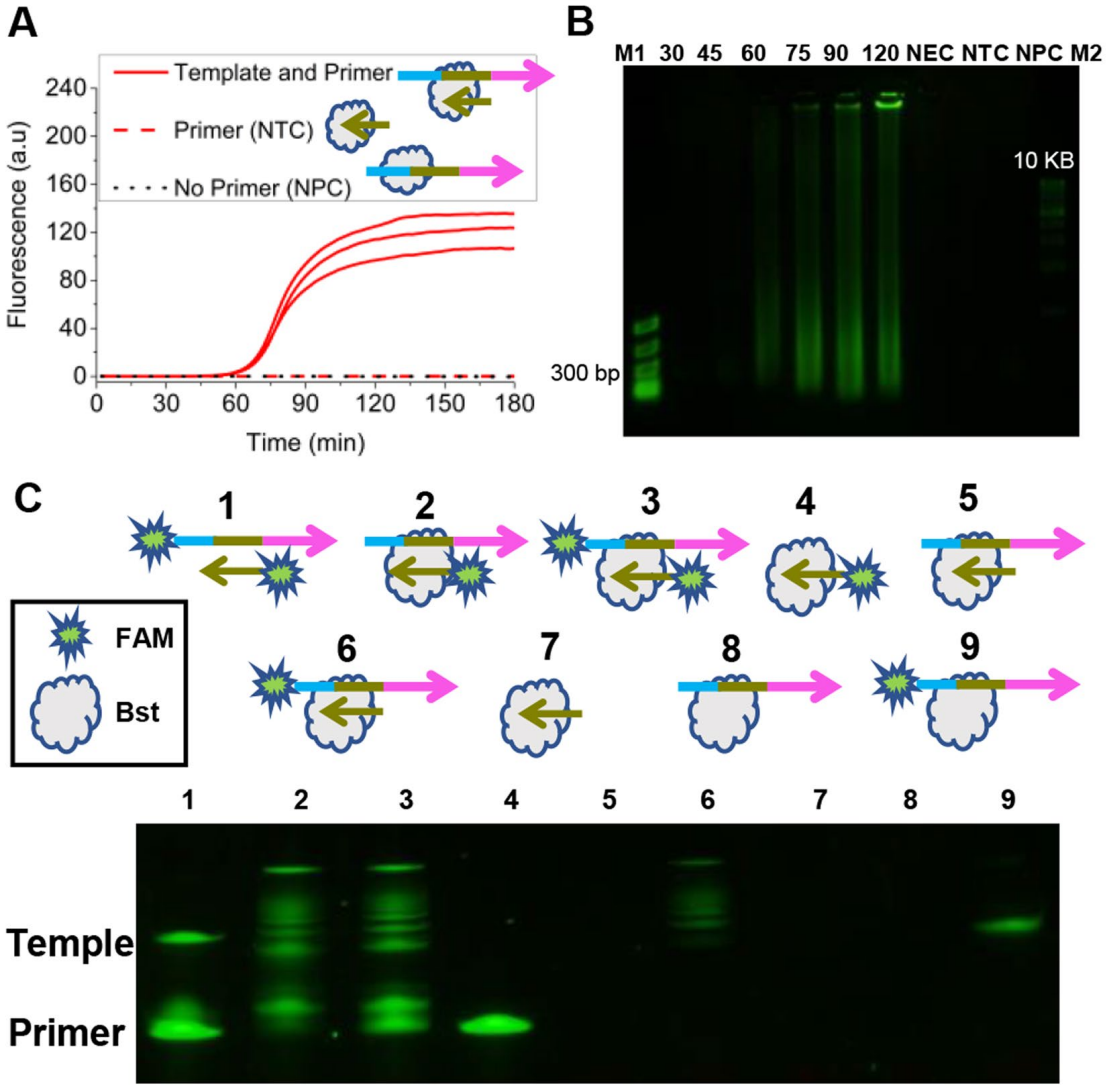
Real-time fluorescence and electrophoresis analysis of UIMA. All reactions shared the same primer (RL) or template (F*R*), and were incubated for 180 min. The sequences of RL and F*R* were shown in Table S1. (**A**) The results of real-time fluorescence obtained from the reactions that contained 10 nM template, 1.6 μM primer, and 3.2 U *Bst* WS DNA polymerase at 63 °C for 180 min. Each test was in triplicate. (**B**) Time course of the UIMA assay. 2.5% agarose gel electrophoresis shows the products of UIMA. The assay time was varied from 30–120 minutes as indicated above each lane. M1 and M2: DNA marker; NEC, NTC and NPC were all incubated for 120 min. Exposure time is 5 s. (**C**) Extension status of template and primer. Template and primer were labeled with the FAM fluorophore. 1: reaction with FAM-labeled primer and template but no *Bst*; 2: with FAM-labeled primer, non-labeled template, and *Bst*; 3: with FAM-labeled primer and template and *Bst*; 4: with FAM-labeled primer and *Bst* but no template; 5: with non-labeled primer and template, and *Bst*; 6: with non-labeled primer, FAM-labeled template, and *Bst*; 7: with non-labeled primer and *Bst* but no template; 8: with non-labeled template and *Bst* but no primer; 9: with FAM-labeled template and *Bst* but no primer. All the reactions were incubated at 63 °C for 180 min. Their products were analyzed by 17% denatured polyacrylamide gel electrophoresis (DPAGE). NEC (no-enzyme control): the reaction without *Bst* 2.0 WS DNA polymerase. NTC (no-template control): control reaction just lacked the template; NPC (no-primer control): control reaction lacked primer RL. Horizontal arrows denoted the 5′-3′ direction of sequences. Exposure time is 5 s. The full-length gels are presented in Supplementary Figure S1.

As shown in Fig. 1A, within 180-min reaction time, an exponential fluorescence change was obviously indicated in the reactions with both template and primer, whereas not presented in NTCs and NPCs. As a further confirmation, a time course for product was analyzed by agarose gel electrophoresis (Fig. 1B). When the incubation time reached 60 min, larger DNA fragments than the template started to accumulate. As time went on, an increasing number of large fragments were produced and even full of the lanes. At 120 min, fragments with over 10 kb were clearly observed. However, this phenomenon didn’t appear at the controls of NTC, NPC, and NEC. Regarding this result, UIMA was undoubtedly a template-dependent reaction which could generate products with various sizes.

On the other hand, whether UIMA was an amplification reaction was also considered. Figure 1C revealed the extension status of FAM (6-carboxyfluorescein)-labeled template or primer through the denaturing polyacrylamide gel electrophoresis (DPAGE). Without *Bst* WS, both labeled template and primer kept at the original sizes (**lean 1**). Once adding the polymerase, however, labeled fragments with different sizes were clearly indicated in the reactions containing only FAM-labeled primer (**lean 2**), only FAM-labeled template (**lean 6**), and both (**lean 3**). Accordingly, both primer and template were amplified in UIMA and the polymerase was indispensable to initiate reaction. Moreover, the labeled primer was not extended in the reaction without template (**lean 4**), which further verified that UIMA by *Bst* WS was template-dependent. Summarily, though using one primer, UIMA was certainly a template-dependent amplification reaction.

### LIMA was just a specific form of UIMA

**Figure 2:**
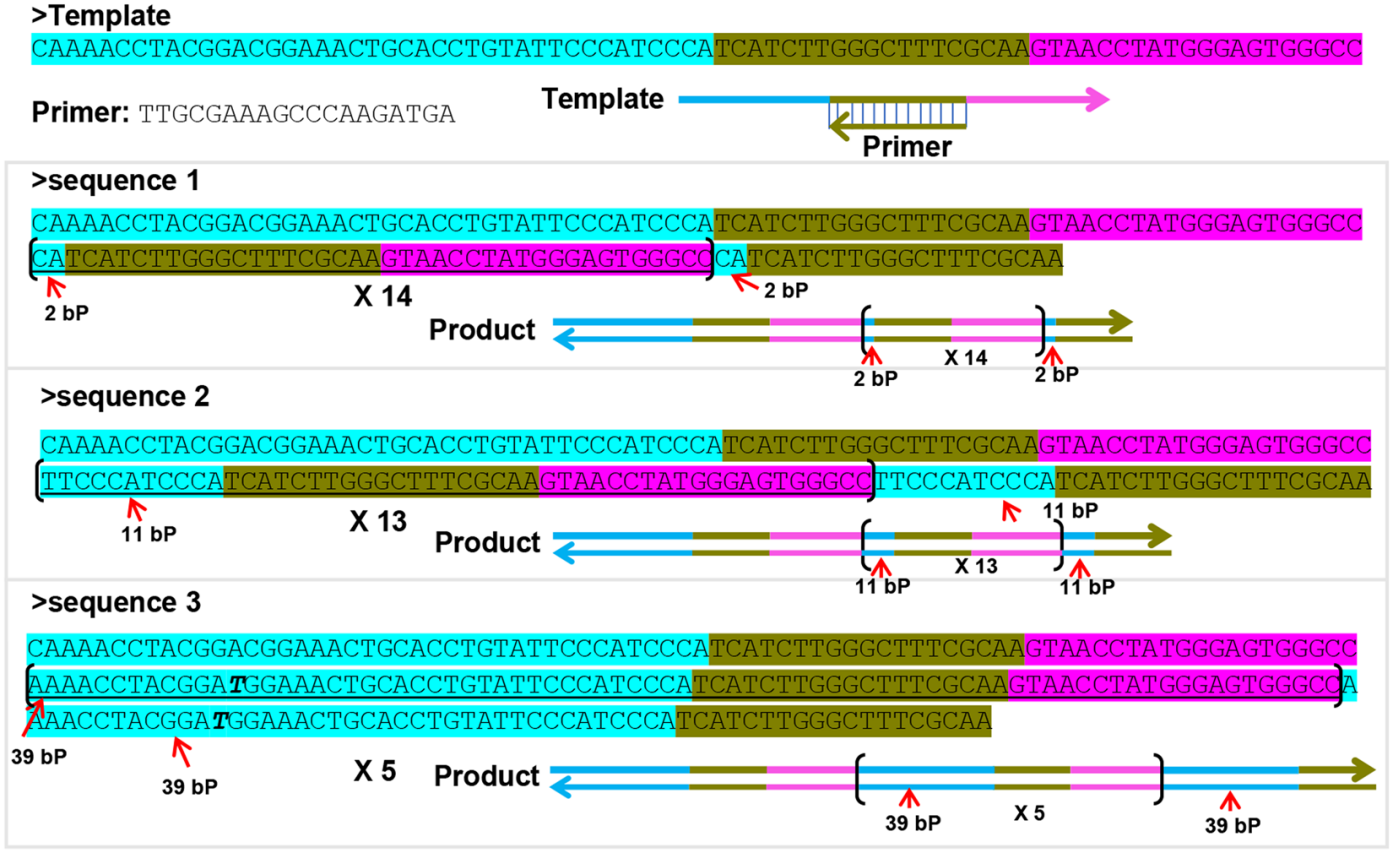
Sequence analysis of UIMA products. The reaction was incubated at 63 °C for 180 min, then the product was cloned into the T-vector and sequenced. Sequence 1, 2, 3 were the three kinds of sequencing results of one UIMA reaction. The braces showed the position of the repeating units. The numbers under the braces showed the number of consecutive repeats in the sequence. The italics and bold bases in the sequence indicated mutations. Horizontal arrows denoted the 5′-3′ direction of sequences.

In order to figure out the sequence composition, the products of UIMA with specific sizes were purified and then cloned into a T-vector for sequencing analysis. As depicted in Fig. 2, its products were multimeric DNAs with repeated sequences derived from template and the repeated units are changeable. But these units had similarity in sequence composition. The upstream part (**blue**) of the unit randomly copied from the 5′ end of template sequence, and its downstream part (**dark yellow** and **pink**) completely replicated the primer-binding site and 3′ end sequences (Fig. 2). Although they varied from different multimeric DNA products, these units remained unchangeable within one specific amplicon. Thus, through sequencing analysis, we found UIMA’s product displayed the same sequence composition as that of LIMA^15^, evidencing that LIMA was just a specific form of UIMA.

### Flanking sequences of the primer recognition site influenced UIMA’s efficiency

**Figure 3:**
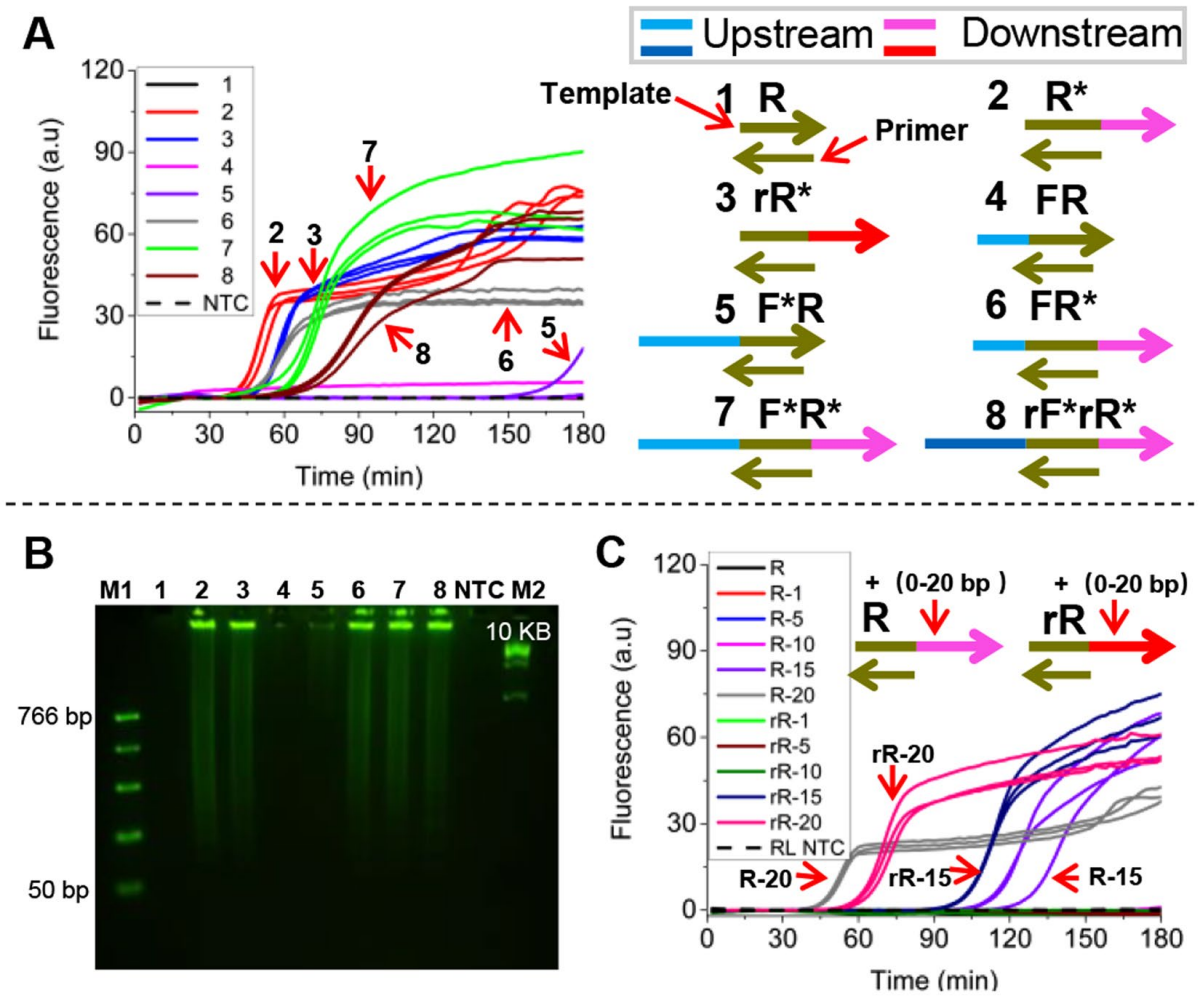
Flanking sequences (FS) of the recognition site influenced on the efficiency of UIMA. (**A**) Real-time fluorescence change in reactions with the same primer (**RL**) but different templates when incubated at 63 °C for 180 min. 1: template only with primer site (**R**); 2: with primer site and 3′ end FS (R*); 3: with primer site and changed 3′ end FS (rR*); 4: with primer site and 5′ end FS (**FR**); 5: with primer site and lengthened 5′ end FS(F*R); 6: with primer site and FSs at both 3′ and 5′ ends (FR*); 7: with primer site, FS at 3′ end, and lengthened FS at 5′ end (F*R*); 8: with primer site, FS at 3′ end, and changed FS at 5′ end (rF*rR*); NTC: no-template control. No-primer controls (NPCs) were shown in Fig. S1. Detailed sequences were shown in Table S1. (**B**) Product analysis of (**A**) by 2.5% agarose gel electrophoresis. M1 and M2: DNA Marker. Exposure time is 5 s. The full-length gels are presented in Supplementary Figure S4. (**C**) Real-time fluorescence change in reactions using templates with different lengths of 3′ end FSs. R: template only with primer site; R-1 (5, 10, 15, and 20): R templates with 1, 5, 10, 15, and 20 bp sequences added at 3′ end; rR-1 (5, 10, 15, and 20): R templates with 1, 5, 10, 15, and 20 bp changed sequences added at 3′ end; RL: the used primer; NTC: no-template control. NPCs were shown in Fig. S1. Horizontal arrows denoted the 5′-3′ direction of sequences. Each reaction was in triplicate.

According to the results above, the repeated units in each product sequence partially copied from the template. Thus, we speculated that flanking sequences of the recognition site was likely to be associated with UIMA’s efficiency. As shown in Fig. 3A, for any template with downstream flanking sequence at 3’ end, UIMA was initiated within 60 min and performed high efficiency (**Reaction 2, 3**, and **6–8**). What’s more, when the flanking sequence changed (**Reaction 3** and **8**), high efficiency was still achieved. For the upstream flanking sequence at 5′ end, it could influence the reaction speed, and the rapidest UIMA was achieved by using the template without upstream flanking sequence (**Reaction 2**). However, for those templates without downstream flanking sequence at 3′ end, UIMA performed low efficiency (**Reaction 1, 4**, and **5**). Also, their NPCs and NTCs were all difficult to run the UIMA reactions (Figs 3A, S2A and B). Consequently, it was clear that downstream sequences, namely 3′ overhang of the complex formed when primer recognizes the site, seriously affected amplification efficiency, which was further confirmed by the results of agarose gel electrophoresis (Fig. 3B). Similarly, all the amplified products were multimeric DNAs with the template sequence or part of it repeated (Fig. S3).

Moreover, we screened the overhangs with various sizes. As depicted in Fig. 3C, UIMA only occurred when template had an overhang with at least 15 nucleotides, so was in those with changed overhang sequences. Figure 3C also revealed that longer overhangs could mediate rapider UIMA. Certainly, their NPCs and NTCs were all negative. (Fig. S2C).

### UIMA was achieved by the strand-displacing DNA polymerases with RT activities

**Figure 4:**
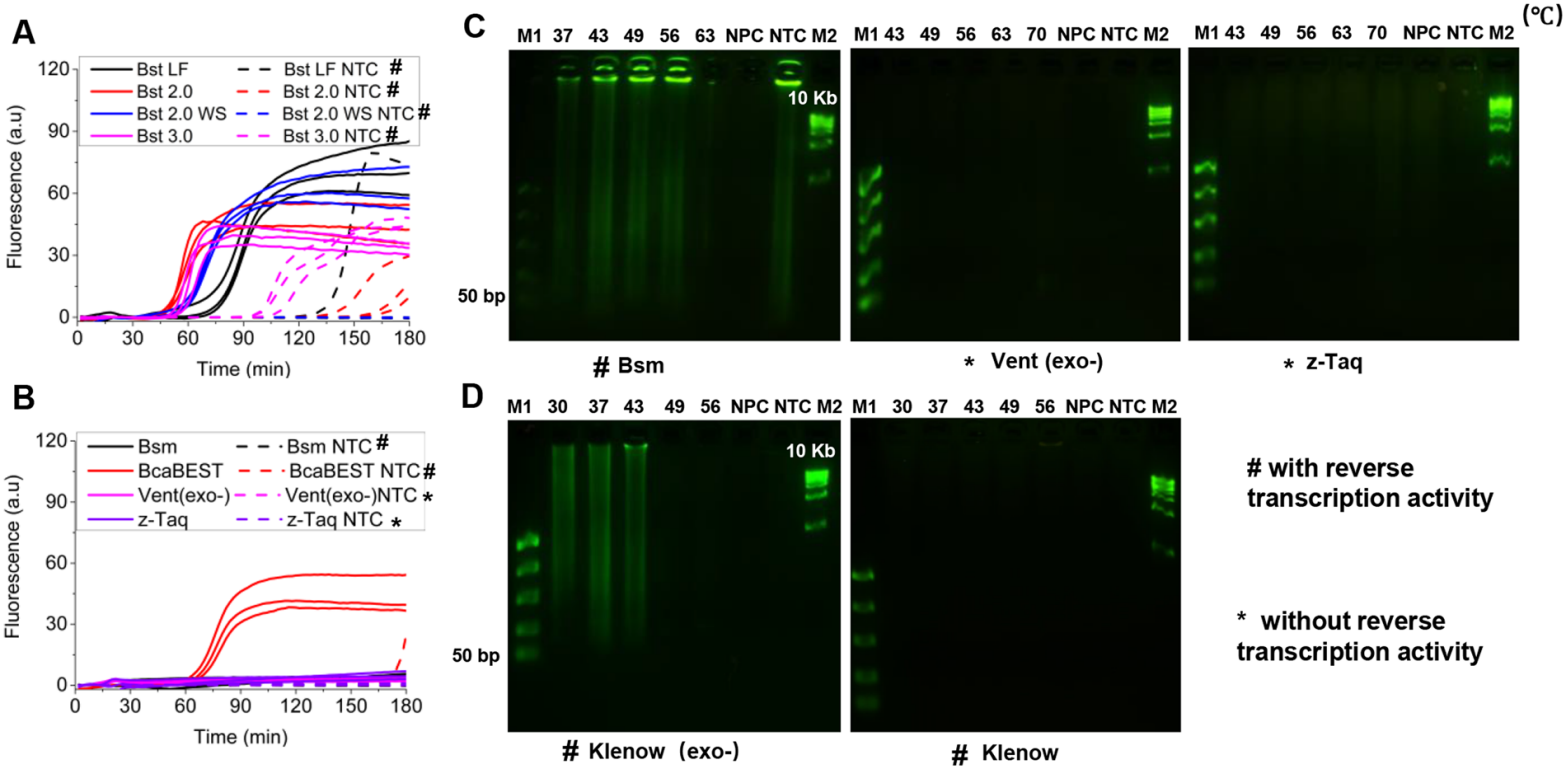
Verification of UIMA using different DNA polymerases. All reactions shared the same primer (RL) and template (F*R*) and were incubated for 180 min. The sequences of RL and F*R* were shown in Table S1. (**A**) Real-time fluorescence change in reactions using a series of *Bst* DNA polymerases (*Bst* LF, *Bst* 2.0, *Bst* 2.0 WS, and *Bst* 3.0) at 63 °C. No-primer controls (NPCs) were shown in Fig. S5. (**B**) Real-time fluorescence change in reactions using non-*Bst* polymerases (Bsm, BcaBEST, Vent(exo-), and z-Taq) at 63 °C. No-primer controls (NPCs) were shown in Fig. S5. (**C**) Temperature gradients assay for the products of reactions using the polymerases with negative results in (**B**). The products were analyzed by 2.5% agarose gel electrophoresis. NTC and NPC for Bsm were performed at 56 °C. NTCs and NPCs for Vent (exo-) and z-Taq were performed at 63 °C. The groping of gels cropped from different gels. Exposure time is 5 s. (**D**) Temperature gradients assay for the products of reactions using the polymerases of Klenow(exo-) and Klenow. The products were analyzed by 2.5% agarose gel electrophoresis. Their NTCs and NPCs were performed at 43 °C. M1 and M2: DNA Marker. NTC: no-target control; NPC: no-primer control. The groping of gels cropped from different gels. Exposure time is 5 s. The full-length gels are presented in Supplementary Figure S7.

As an enzymatic reaction, UIMA depended on the activity of *Bst* WS. Thus, it was necessary to investigate whether other *Bst* DNA polymerase types were still workable for UIMA. As provided by New England Biolabs, *Bst* DNA polymerase large fragment (*Bst* LF), *Bst* 2.0 DNA polymerase (*Bst* 2.0), and *Bst* 3.0 DNA polymerase (*Bst* 3.0) were used to replace *Bst* WS. As shown in Fig. 4A, UIMA successfully happened with all of these polymerase types, whereas not in the NPCs (Fig. S5A). Compared to *Bst* WS, however, these polymerases were not helpful to achieve robust NTCs, especially the *Bst* 3.0 which could trigger fast template-independent reactions (Fig. 4A).

Apart from *Bst* polymerase, other DNA polymerases with strand displacement activities were also evaluated, such as BcaBEST DNA polymerase (BcaBEST), Vent (exo-) DNA polymerase (Vent (exo-)), z-Taq DNA polymerase (z-Taq) and Bsm DNA polymerase (Bsm). Among them, only the BcaBEST stimulated UIMA at 63 °C and its NTC and NPC didn’t cause the reaction (Figs 4B and S5B). Regarding that 63 °C might be not proper for the polymerases above with negative results, other temperatures were therefore studied. As displayed in Fig. 4C, Bsm was capable of stimulating UIMA in the range of 37 °C to 56 °C, but Vent (exo-) and z-taq still disabled the stimulation of UIMA no matter what temperature was tested. Interestingly, as described by the vendors, *Bst* and BcaBEST actually shared a common feature of implementing RT activity. As to Bsm, it was also evidenced to have a RT activity (Fig. S6). But Vent (exo-)^27^ and z-taq didn’t have RT activities. It thereby suggested that efficient UIMA was caused only by those strand-displacing DNA polymerases with RT activities. To further verify it, two strand-displacing DNA polymerases with RT activities, namely klenow DNA polymerase (klenow) and klenow (exo-) DNA polymerase (klenow (exo-)) were employed to develop UIMA. As shown in Fig. 4D, UIMA only presented when using klenow (exo-) from 30 °C to 43 °C, indicating that the 3′–5′ exonuclease activity of klenow performed inhibition for UIMA, though possessing RT activity^27^. But RT activity was essential to achieve UIMA, since the polymerase of Vent (exo-) lacking both 3′–5′ exonuclease and RT activities still failed to simulate reaction (Fig. 4C).

Furthermore, three commonly used reverse transcriptases of AMV, MLV, and MHM were also investigated. Figure S8 revealed that only MHM successfully stimulated UIMA when reacting at 37 °C, hinting a new finding that MHM also possessed high DNA polymerase activity. The reason why UIMA hardly happened using AMV and MLV was likely that they performed poor strand displacement activities, which was also the reason why RT-LAMP usually required two enzymes of *Bst* and reverse transcriptase^28,29^. In addition, we also experimented through combining the DNA polymerase without RT activity (z-Taq) and the reverse transcriptases of AMV, MLV, and MHM, respectively. As shown in Fig. S8, z-Taq coupled with AMV or MLV was not able to initiate the reaction, suggesting that a compensation using reverse transcriptase for the DNA polymerase short of RT activity was ineffectual. This result also implied that, to stimulate UIMA, DNA polymerase activity and RT activity could not be separated from a strand-displacing DNA polymerase.

According to the results of sequencing analysis by using T-vector (Fig. S9), the products from BcaBEST-, Bsm- and Klenow (exo-)-based UIMAs were similar to those from *Bst*-based UIMAs, but the Klenow (exo-) could introduce extra non-template sequences to the products. Moreover, the products of MHM were hard to be cloned into the T-vector. It meant that pure reverse transcriptase-mediated UIMA was probably different from those using the strand-displacing DNA polymerase with RT activities, which deserved being investigated in the future study. Anyway, our findings demonstrated that strand-displacing DNA polymerase with RT activities stimulated efficient UIMA, but essentially without 3′–5′ exonuclease activities. Unfortunately, after a long-time incubation, products were also indicated in NTCs with *Bst* LF, *Bst* 2.0, *Bst* 3.0 and Bsm (Fig. 4A and C). Through sequencing their products, their sequences typically consisted of primer sequences and other additional sequences (Fig. S10). This additional sequence was added probably due to DNA polymerases’ *ab initio* synthesis which usually required rather long incubation^30^.

### UIMA performed high sensitivity, high specificity and universality

**Figure 5:**
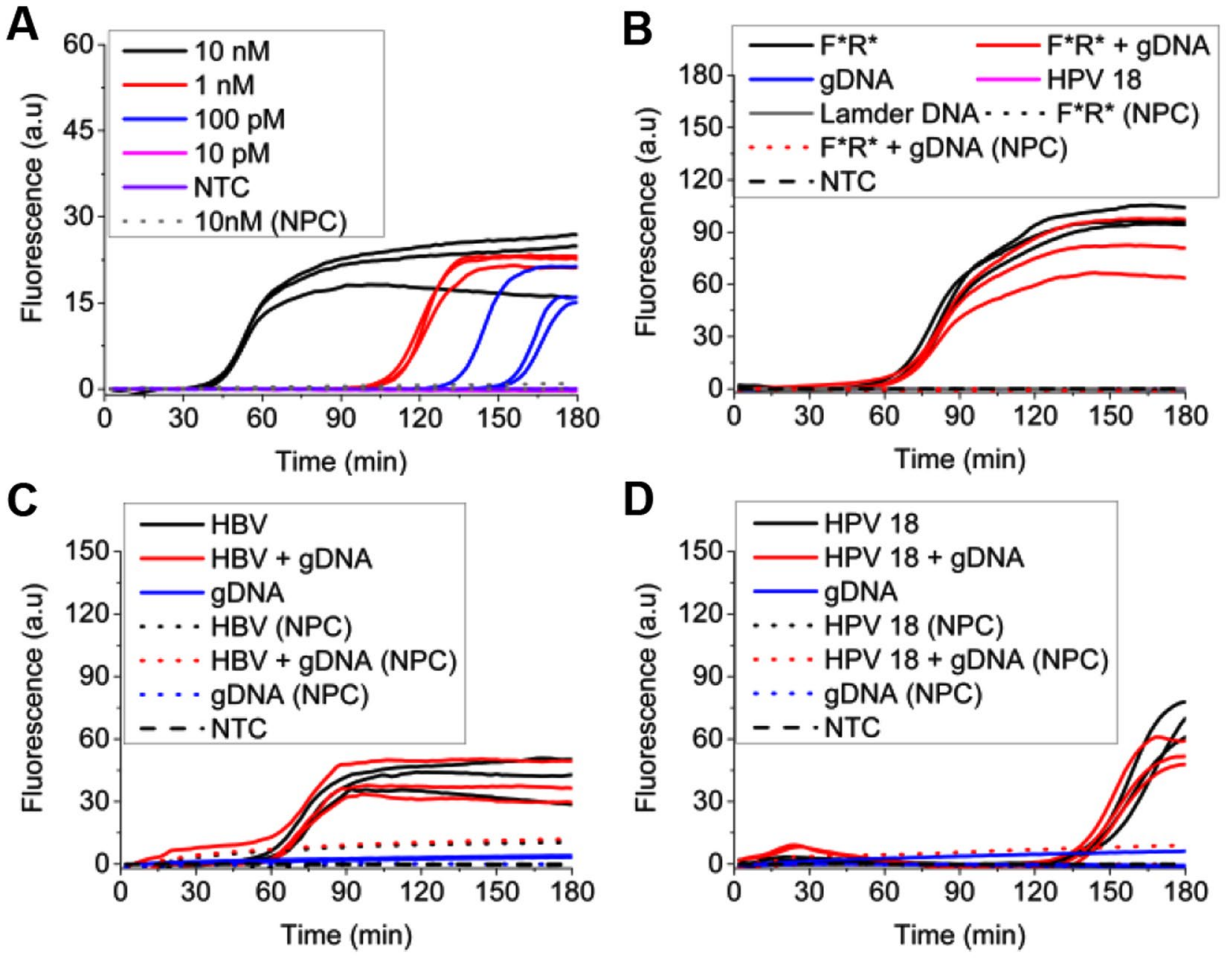
Sensitivity, specificity, universality assay of UIMA. All reactions were performed at 63 °C for 180 min. (**A**) Sensitivity of UIMA using 1.6 μM RL primer and different concentration (from 10 nM to 10pM) of F*R* template with 10-fold dilution. (**B**) Specificity of UIMA with 1.6 μM RL primer to assay different templates including 10 nM F*R*, 10 nM synthesized ssDNA partially obtained from genomic DNA of HPV 18 subtype (HPV 18), 1 ng Lamder DNA, 2 ng human genomic DNA (gDNA), and the mixtures of 10 nm F*R* and 2 ng gDNA. (**C**) and (**D**) Universality of UIMA with 1.6 μM corresponding primers to detect 10 nM templates of HBV DNA, HPV 18 DNA, and their mixtures with gDNA. HBV DNA was a synthesized ssDNA from a part of genomic HBV DNA.; NTC: no-template control; NPC: no-primer control. Each reaction was in triplicate. Detailed sequences were shown in Table S1.

As shown in Fig. 5A, the sensitivity of UIMA reached down to 100 pM templates at 180-min reaction. Although UIMA was one primer-mediated amplification, its sensitivity was comparable to that of two primers-based LIMA^15^. To analyze specificity, as shown in Fig. 5B, target template (F*R*) was replaced by human genomic DNA (gDNA), non-target synthesized ssDNAs from genomic HPV 18 or lambda DNA, and the mixture of F*R* and gDNA. Exponential fluorescence signal was only achieved by the target template while not for others (Fig. 5B), which was further confirmed by agarose gel electrophoresis assays (data not shown). In Fig. 5C and D, target templates from HBV and HPV18 and their mixtures with gDNA were also successfully amplified by using their corresponding primers. Clearly, their NTCs and NPCs all failed to react (Fig. 5). Also, their products were multimeric DNAs with repeated sequences derived from template (Fig. S11). As a simple INAA, UIMA therefore possessed high sensitivity, high specificity and universality.

### A plausible mechanism of UIMA

**Figure 6:**
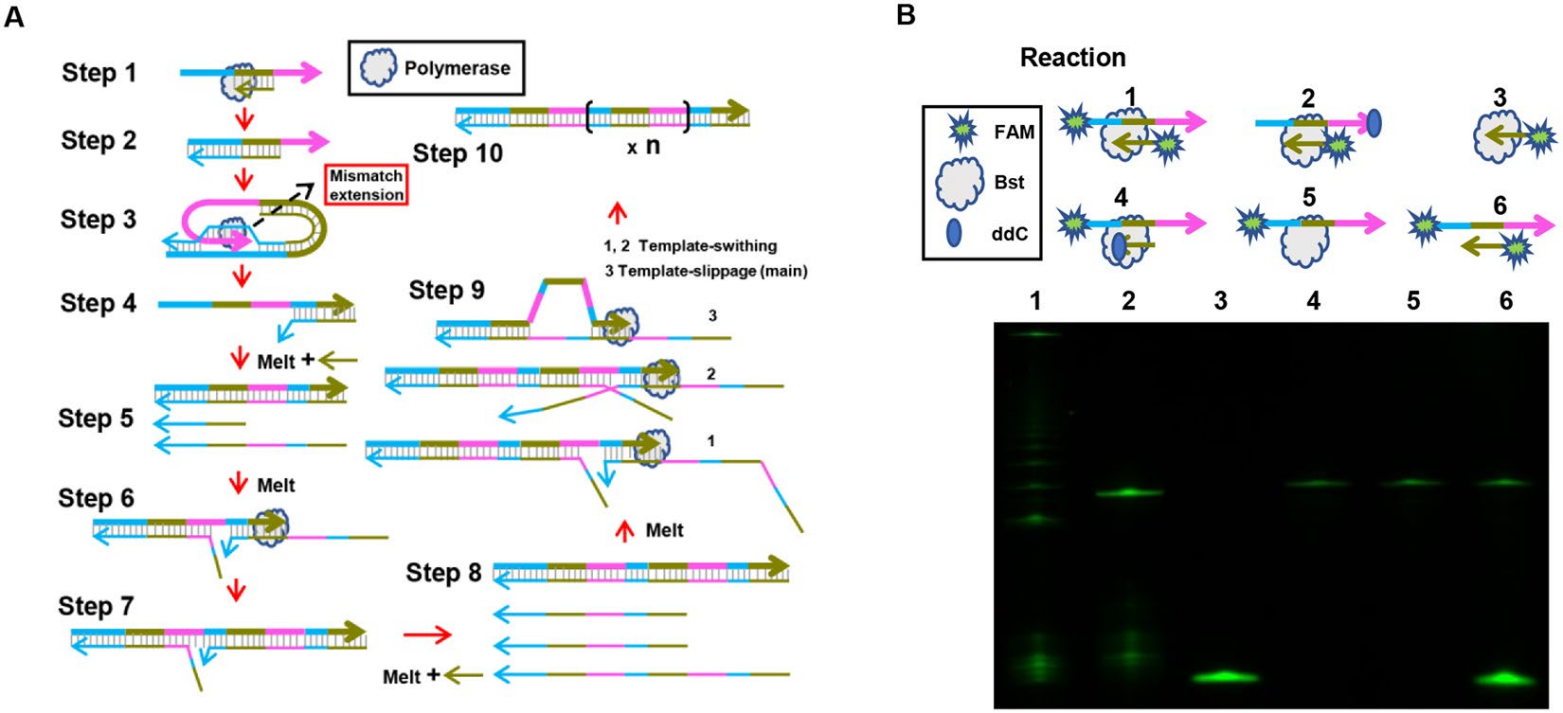
Proposed mechanism of UIMA and 3′ end block assay. (**A**) Schematic showing the mechanism of UIMA. (**B**) Extension status of template or primer with ddC-based 3′ end block.1: reaction with FAM-labeled primer, FAM-labeled template, and Bst; 2: with FAM-labeled primer, ddC-labeled template, and Bst; 3: with FAM-labeled primer and Bst; 4: with ddC-labeled primer, FAM-labeled template, and Bst; 5: with FAM-labeled template, and Bst; 6: with FAM-labeled primer, FAM-labeled template, but no Bst; All reactions shared the same primer (RL) and template (F*R*) and were incubated at 63 °C for 180 min. The sequences of RL and F*R* were shown in Table S1. Their products were analyzed by 17% denatured polyacrylamide gel electrophoresis (DPAGE). Horizontal arrows denoted the 5′-3′ direction of sequences. Exposure time is 5 s. The full-length gels are presented in Supplementary Figure S12.

According to the conclusions above, a plausible mechanism of UIMA was proposed in Fig. 6A. First, the primer annealed to the template and extended (**step 1** and **2**). Then, free 3′ overhang bended and mismatched the primer’s extension strand. Meanwhile, the strand-displacing polymerase with RT activity bound this specific structure to initiate the extension of 3′ overhang (**step 3**). Since this step was very fast and probabilistic, high concentration of template in the reaction was indispensable to guarantee high triggering probability. After extending, a template with two primer binding sites was produced (**step 4**). With the aid of primer, this new template became dsDNA (**step 5**). In a dynamic process, partial matching occurred between sense and antisense strands (**step 6**), producing the strand with three primer-recognizing sites (**step 7**). Next, more antisense strands with primer sequences were produced and it increased the opportunities for 3′ ends of sense strands annealing to the antisense strands (**step 8** and **9**). Owing to an increasing number of long repeated sequences generated, the reaction performed an exponential amplification, causing the products accumulated (**step 10**). Mixed occurrence of dsDNA melting, template switching, and template slippage started from **step 4** and facilitated the reaction, because of the dynamic denaturation and renaturation of dsDNA at 63 °C and especially under a high concentration of betaine denaturant^31^.

To indirectly prove the proposed principle, 2′,3′-dideoxynucleotide (ddC)- or FAM-labeled primer and template were used to develop UIMA. As shown in Fig. 6B, UIMA was completely inhibited in the reactions with ddC-labeled template or primer at their 3′ ends (**Reaction 2** and **4**), demonstrating that free 3′ overhang of template was very essential to trigger the reaction through mismatching the primer’s extension strand at the beginning. However, to thoroughly testify this mechanism, capturing real-time formation of the structure at **step 3** should be of great concern for the future study. On the other hand, since the repeated units always kept stable within the same product sequence, UIMA might have the similar mechanism as the extension of telomeres in which DNA translocation often appeared in the long strand with repeated sequences^32^.

## Discussion

Presently, using one primer to synthesize multimeric DNA, namely the DNA with target sequence repeated or partially repeated, is only observed in rolling circle amplification (RCA) which is established based on a template of circular oligonucleotide^33–35^. Whereas, making the sequence of linear template incorporated into multimeric DNA has to depend on the use of at least two specific primers^15^. In general, using primers to amplify linear template can produce fragments with prescient size, typically in PCR. But, due to the employment of DNA polymerases with strand displacement activities in INAAs, predicating the size of product fragments is complicated. For example, two PCR-like primers-based LIMA produces multimeric dsDNA, through amplifying linear template by *Bst* DNA polymerase^15^. In this study, our findings indicate that multimeric DNA can be produced using the linear template, one primer, and a strand-displacing DNA polymerase with RT activity. In other words, LIMA is just a specific form of UIMA.

In the theory of strict base pairing-priming extension, UIMA cannot yield large fragments but only the short primer-template dsDNA complex with a 3′ end overhang (seen in **Step 2** in Fig. 6). However, UIMA indeed takes place with large-size fragments produced. Since no circular template or uniquely designed primer is used, an unusual substrate structure is likely formed by the complex. As reported previously, short DNA can bend and stabilize the looped state in the buffer containing high concentrations of Mg^2+^ (e.g. 10 mM)^36–38^. In UIMA’s buffer, Mg^2+^ is 8 mM close to 10 mM. Consequently, the primer-template complex tends to be in the looped state. On the other hand, high concentration of Mg^2+^ enhances the DNA mismatch’s probability. Due to the mismatch of 3′ end overhang to primer-extended strand, specific substrate structure is therefore yielded (seen in **Step 3** in Fig. 6). By labeling the primer and template, we prove that the 3′ ends of primer and template both function during amplification. Besides, the 3′ end overhang at the primer-template complex plays an essential role in achieving efficient UIMA, because unbinding ssDNA in the ds complex might perform a strong capability of mediating mismatch extension, compared to dsDNA part. So, when using ddC to block the 3′ end of template or primer, UIMA can be entirely inhibited. Given this, it is sure that 3′ end mismatching is equally important as 3′ end extension in UIMA.

Apart from DNA blending and mismatch extension, our findings also indicate that the used strand-displacing DNA polymerase with RT activity is a key factor to realize efficient UIMA, such as *Bst*, BcaBEST, Bsm, and Klenow (exo-). These polymerases are not highly fidelity and most lack 3′–5′ exonuclease activities, thereby hardly correcting the mismatch extension. For this reason, UIMA is not observed when using Klenow with 3′–5′ exonuclease activity. Also, these polymerases have terminal transferase activity through which few bases (e.g. adenine) can be randomly added into the 3′ ends of primer-extended strands. Because of this feature, UIMA’s products can be cloned into T-vectors (e.g. pEASY-T5 vector system) for sequencing analysis.

In this study, we also find that the reverse transcriptase of MHM can stimulate the UIMA reaction. Since reverse transcriptase employs the same domain to function RNA-dependent and DNA-dependent DNA polymerase activities, MHM in UIMA may serve as a telomerase-like role^32,39,40^. Resembling telomerase, MHM may add a repeat sequence to the 3′ end of the template and form an unusual substrate structure. Therefore, reverse transcriptase-based UIMA may influence the veracity of RT-INAAs, for example, RT-LAMP, RT-CPA, and RT-IMSA.

If taking ssDNA template as a unique primer, UIMA becomes a specific dimer-based nonspecific amplification, although it is template-dependent. Thus, investigating UIMA in existing INAAs is helpful to address nonspecific amplification thoroughly, for example, through removing the strand-displacing DNA polymerase’s RT activity and increasing its fidelity. Besides, UIMA can explain many phenomena in existing INAAs. For example, in single-crossing CPA the unintended large-size products are likely caused by UIMA^10^. Loop primers-contained LAMP achieves rapid amplification^18^, likely due to the side reaction of UIMA. Nevertheless, as a simple INAA, UIMA also performs high sensitivity, specificity and universality for the detection of target sequences.

In conclusion, UIMA by the strand-displacing DNA polymerases with RT activities provides a new insight for deeply understanding INAA and opens a new avenue for thoroughly addressing nonspecific amplification.

## Methods

### General information

*Bst* DNA polymerase Large fragment (*Bst* LF), *Bst* 2.0 DNA polymerase (*Bst* 2.0), *Bst* 2.0 WarmStart DNA polymerase (*Bst* 2.0 WS), *Bst* 3.0 DNA polymerase (*Bst* 3.0), Klenow fragment polymerase (Klenow), Klenow fragment exo- polymerase (Klenow (exo-)), Vent exo- DNA polymerase (Vent (exo-)), and dNTP Mix were purchased from New England Biolabs. Bsm DNA polymerase, Maxima H Minus Reverse Transcriptase (MHM), Nuclease-Free Water were purchased from Thermo Fisher Scientific. BcaBEST RNA PCR kit Ver.1.1, z-Taq, RTase M-MLV (RNase H-), Reverse Transcriptase XL (AMV) were purchased from Takara. All synthetic oligonucleotides were purchased from Sangon Biotech (Shanghai, China) **(**Table S1 **)**.

### Real-time fluorescence assay

The real-time fluorescence assay was carried out in a 10 μl reaction mixture containing the following components: 0.4 μl *Bst* 2.0 WS, 1 μl 10 X Isothermal Amplification Buff (which is composed of 20 mM Tris-HCl, 10 mM (NH_4_)_2_SO_4_, 50 mM KCl, 2 mM MgSO_4_, 0.1% Tween 20), 6 mM MgSO_4_, 4% DMSO, 1.6 mM each deoxynucleoside triphosphate (dNTP), 0.6 X SYBR Green I. Then 10 nM DNA template and 1.6 μM primer were added to the tube. Meanwhile, three control tests of no-template control (NTC), no-primer control (NPC), and no-enzyme control (NEC) were set up. A real-time fluorescence detection instrument (ABI 7900HT) was used to acquired signal and the time and temperature for the reaction was set for 180 min at 63 °C. The products were analyzed by electrophoresis in a 2.5% agarose gel (1 X TAE) stained with 1 X SYBR Green I. All the reactions with *Bst* WS were used the same reaction system, and the only difference was the types of template or primer. The fluorescent images of gels were captured through the liquid-crystal tunable filter automatically tuned with 10 or 5 nm increment in a range of 500 nm to 650 nm in a Maestro Ex *IN-VIVO* Imaging System (CRI Maestro, USA). The fluorescence was excited by a blue light at 455 nm with an exposure time of five seconds, and the emitted light was accepted by a large area CCD camera through a 495 nm long-pass filter.

### Template and primer extension

The FAM (6-carboxyfluorescein) was added into the 5′ end of the template (F*R*) or primer (RL). The reaction with FAM-labeled template or primer was incubated at 63 °C for 180 min, and the reaction system was the same as described above. Their products were analyzed by 17% denatured polyacrylamide gel electrophoresis (DPAGE) with 7 M urea to denature dsDNA into single-stranded DNA.

### Cloning and sequencing

The products were electrophoresed in a 2.5% Tris–acetic acid–EDTA (TAE) agarose gel for 40 min. Nucleic acid was stained with SYBR Green I. The nucleic acid was recovery by agarose gel purification kits (ComWin Biotech). Then the products were cloned into pEASY-T5 vector (TransGen Biotech) and sequenced, which was provided by Sangon Biotec (Shanghai, China).

### Confirmation of UIMA by different DNA polymerases

The assay was divided into two parts: one was real-time fluorescence experiment and the other one was temperature gradient experiment. The buffers for real-time fluorescence experiment and temperature gradient experiment were same to *Bst* WS’. But the temperatures of temperature gradient experiments were different from real-time fluorescence experiment (63 °C). The temperature gradient depended on the optimum temperature of the enzyme. The used amount of BcaBEST, Vent (exo-), Z-taq, Bsm, Klenow, or Klenow (exo-) was 3.2 U, the same as that of a series of *Bst* DNA polymerases. But MLV, MHM, and AMV in reaction were 150 U, 150 U, and 15 U, the optimum amounts used in the corresponding reverse transcription reactions. The mixtures were incubated with template (F*R*) and primer (RL) for 180 min.

## Electronic supplementary material

Supplementary Information
