# Supplementary material for: Unusual isothermal multimerization and amplification by the strand-displacing DNA polymerases with reverse transcription activities
Source: Sci Rep. 2017 Oct 24;7:13928. doi: 10.1038/s41598-017-13324-0 (PMC5654958; doi:10.1038/s41598-017-13324-0)
Supplement: Supplementary file 1 — Supplementary Information [file 41598_2017_13324_MOESM1_ESM.pdf]

Supporting Information

Unusual isothermal multimerization and amplification by the strand-displacing DNA polymerases with reverse transcription activities

Guoping Wang[1,2,#], Xiong Ding[1,2,#], Jiumei Hu[1,2], Wenshuai Wu[1,2], Jingjing Sun[1,2] and Ying Mu[1,*]

[1] Research Center for Analytical Instrumentation, Institute of Cyber-Systems and Control, State Key Laboratory of Industrial Control Technology, Zhejiang University, Hangzhou, 310058, P. R. China
[2] College of Life Sciences, Zhejiang University, Hangzhou, 310058, P. R. China

* To whom correspondence should be addressed. Tel: +86 571 88208383; Fax: +86 571 88208383; Email: muying@zju.edu.cn

[#] Co-authorship: These authors contributed equally to this work.

Supplementary Table

Table S1. Sequences of nucleic acids used in this work

Nucleic acids	Sequences (5'-3')
**Tem R**	TCATCTTGGGCTTTCGCAA
**Tem R* (R-20)**	TCATCTTGGGCTTTCGCAAGTAACCTATGGGAGTGGGCC
**Tem rR* (rR-20)**	TCATCTTGGGCTTTCGCAAGAAGTCAATGGTGAGCTAGT
**Tem FR**	GCACCTGTATTCCCATCCCATCATCTTGGGCTTTCGCAA
**Tem FR***	GCACCTGTATTCCCATCCCATCATCTTGGGCTTTCGCAAGTAACCTATGGGAGTGGG
**Tem F*R**	CAAAACCTACGGACGGAAACTGCACCTGTATTCCCATCCCATCATCTTGGGCTTTCGCAA
**Tem F*R***	CAAAACCTACGGACGGAAACTGCACCTGTATTCCCATCCCATCATCTTGGGCTTTCGCAAGTAACCTATGGGAG TGGGCC
**Tem rF*rR*:**	TCACTGAAGCAAGTAGTCACAGCACCTGTATTCCCATCCCATCATCTTGGGCTTTCGCAAGAAGTCAATGGTGA GCTAGT
**Tem R-1**	TCATCTTGGGCTTTCGCAAG
**Tem R-5**	TCATCTTGGGCTTTCGCAAGTAAC
**Tem R-10**	TCATCTTGGGCTTTCGCAAGTAACCTATG
**Tem R-15**	TCATCTTGGGCTTTCGCAAGTAACCTATGGGAGT
**Tem rR-1**	TCATCTTGGGCTTTCGCAAT
**Tem rR-5**	TCATCTTGGGCTTTCGCAAGAAGT
**Tem R-10**	TCATCTTGGGCTTTCGCAAGAAGTCAATG
**Tem rR-15**	TCATCTTGGGCTTTCGCAAGAAGTCAATGGTGAG
**Primer for up sequences RL**	TTGCGAAAGCCCAAGATGA
**HBV**	CTGCTCAAGGAACCTCTATGTTTCCCTCATGTTGCTGTACAAAACCTACGGACGGAAACTGCACCTGTATTCCC ATCCCATCATCTTGGGCTTTCGCAA
**Primer for HBV HBV-RL**	AGTTTCCGTCCGTAGGTTTTG
**HPV-18**	GAAATAGATGGAGTTAATCATCAACATTTACCAGCCCGACGAGCCGAACCACAACGTCACACAATGTTGTGTAT GTGTTGTAAGTGTGAAGCCAGAATTGAGCTAGTAG
**Primer for HPV HPV-18-RL**	CTTCACACTTACAACACATACACA
**Tem F*R*-ddC**	CAAAACCTACGGACGGAAACTGCACCTGTATTCCCATCCCATCATCTTGGGCTTTCGCAAGTAACCTATGGGAG TGGGCC-ddC
**RL-ddC**	TTGCGAAAGCCCAAGATGA-ddC

Supplementary Figure

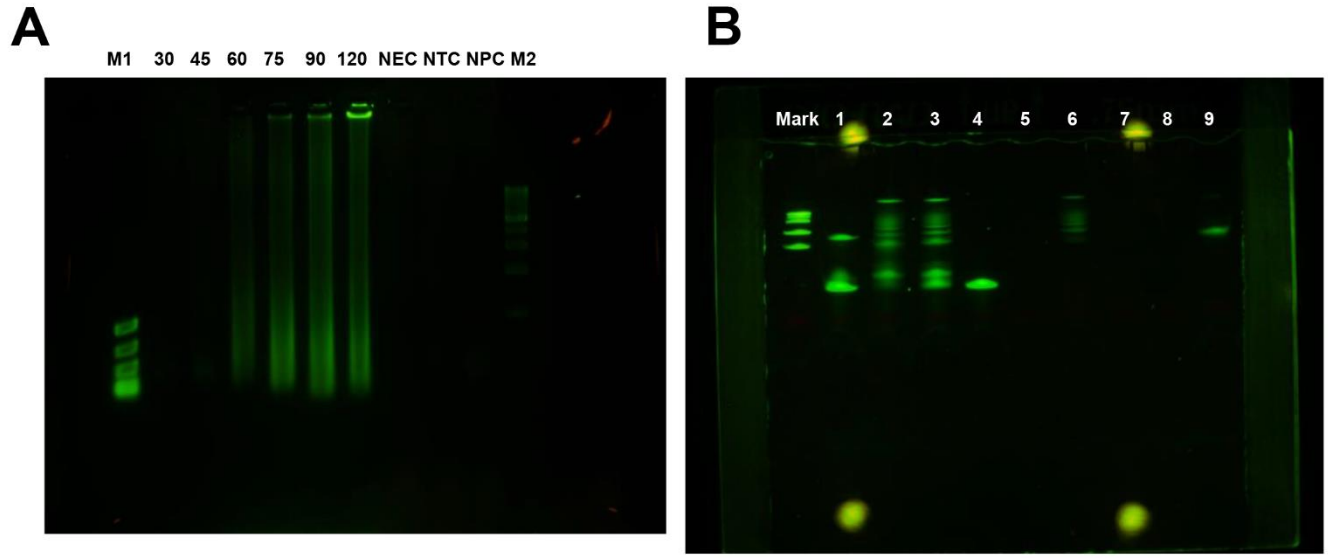

Figure S1. Full-length gels of Figure 1. (A) Time course of the UIMA assay. (B) Extension status of template and primer.

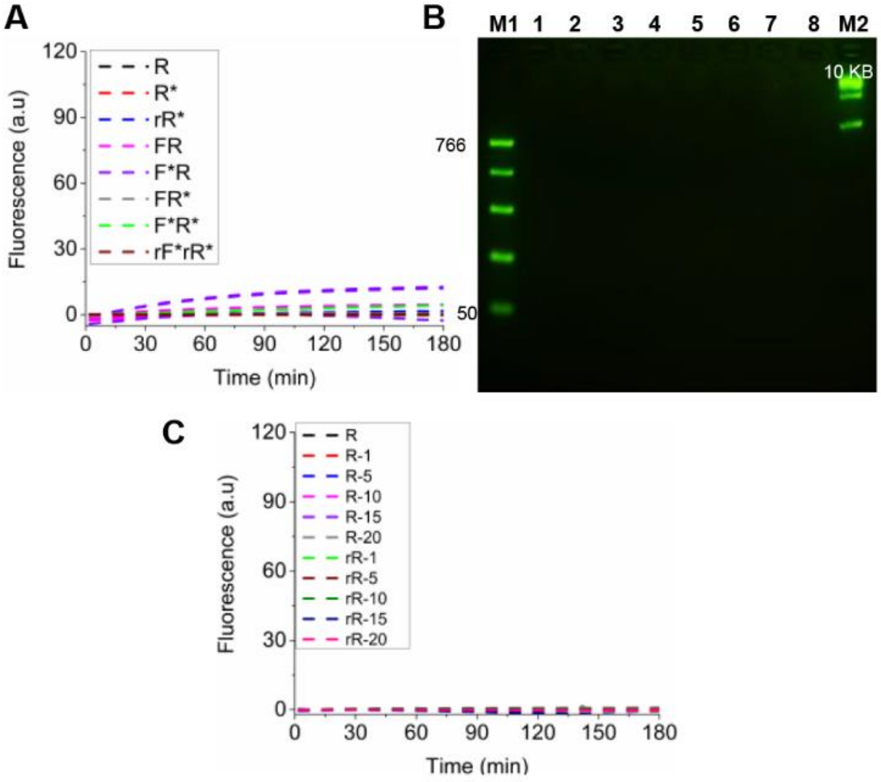

Figure S2. The no-primer control (NPC) assays of UIMAs with different flanking sequences. (A) The NPC reactions in Fig.3A incubated at 63ºC for 180 min. (B) The products of (A) were analyzed by 2.5% agarose gel electrophoresis. Exposure time is 5 s. (C) The NPC reactions in Fig. 3C incubated at 63ºC for 180 min.

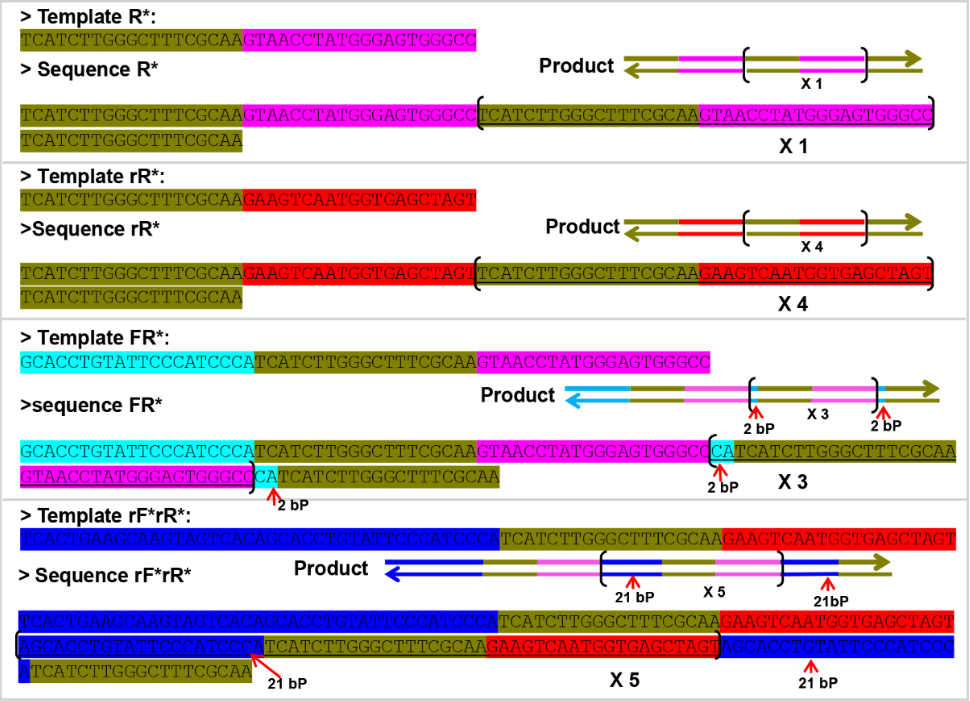

Figure S3. Sequence analysis of UIMA products for the reactions in Fig. 3A. The products amplificated at 63ºC for 180 min were cloned into the T-vector and sequenced. The braces showed the position of the repeating units. The numbers under the underlines showed the number of consecutive repeats in the sequence. Horizontal arrows denoted the 5'-3' direction of sequences.

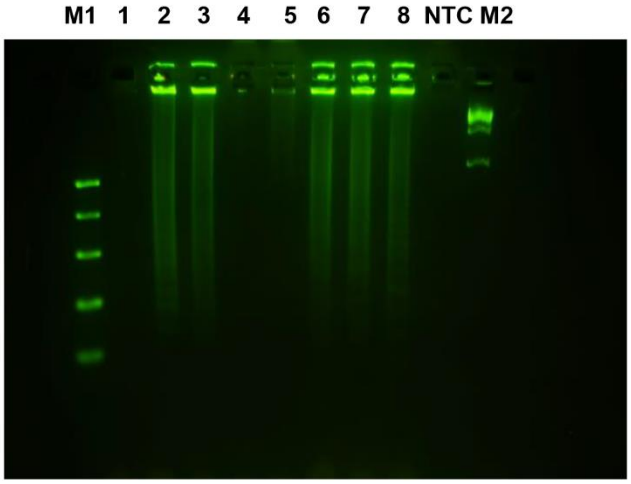

Figure S4. Full-length gels of Figure 3B.

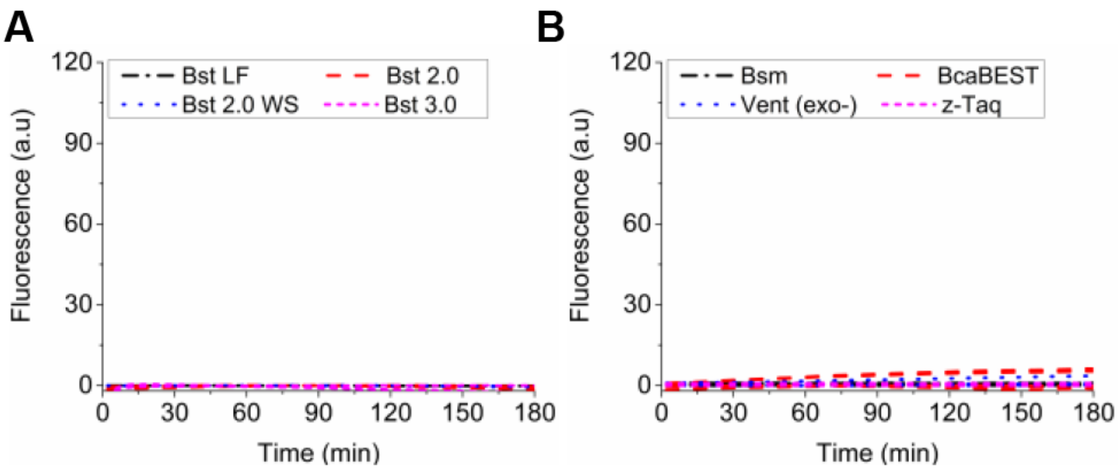

Figure S5. The no-primer control (NPC) assays of UIMAs with different polymerases. (A) The NPC reactions using different *Bst* polymerases incubated at 63ºC for 180 min. (B) The NPC reactions using Bsm, BcaBEST, Vent (exo-) and z-Taq polymerases incubated at 63ºC for 180 min.

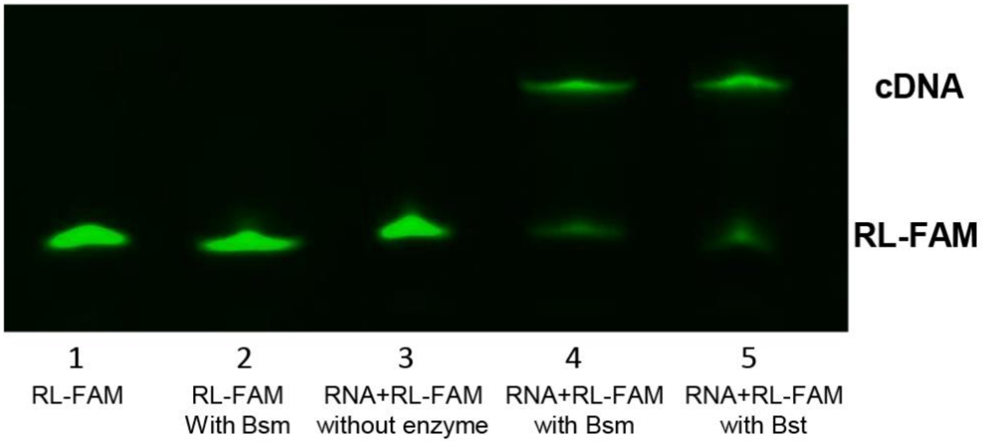

Figure S6. Verification reverse transcriptase activity of Bsm DNA polymerase. Verification reverse transcriptase activities of Bsm by reverse transcription reactions. The labelled primer was hybridized to the RNA and extended with Bsm at 56ºC for 30 min, 98ºC for 5min. Then the products were analysed by 17% denaturing PAGE with 7 M urea, 180V for 40min. Template RNA: CUUGCGAGUGCCCCGGGAGGUCUCGUAGA, Primer-RL-5’ FAM: FAM-TCTACGAGACCTCCCG. Exposure time is 5 s.

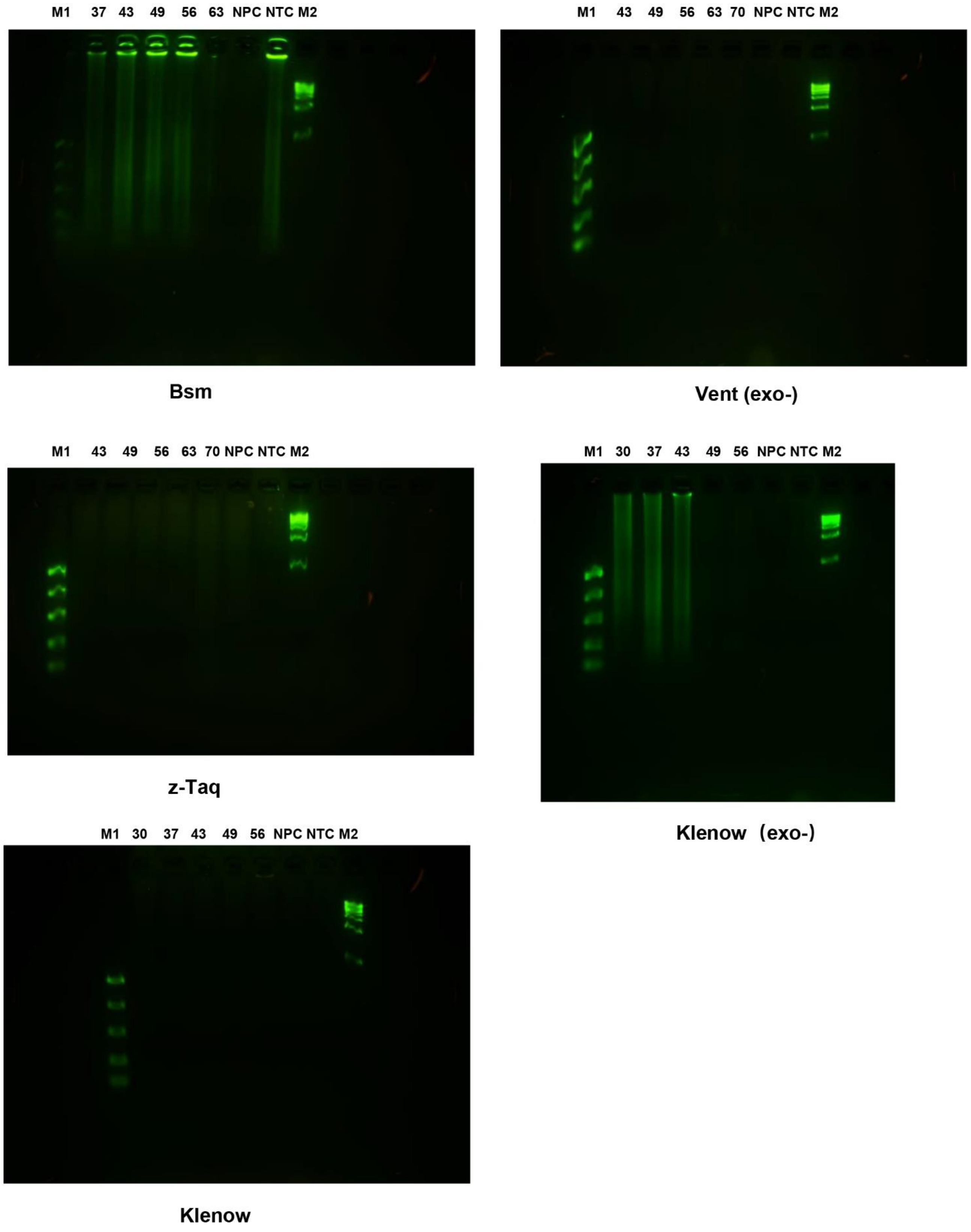

Figure S7. Full-length gels of Figure 4.

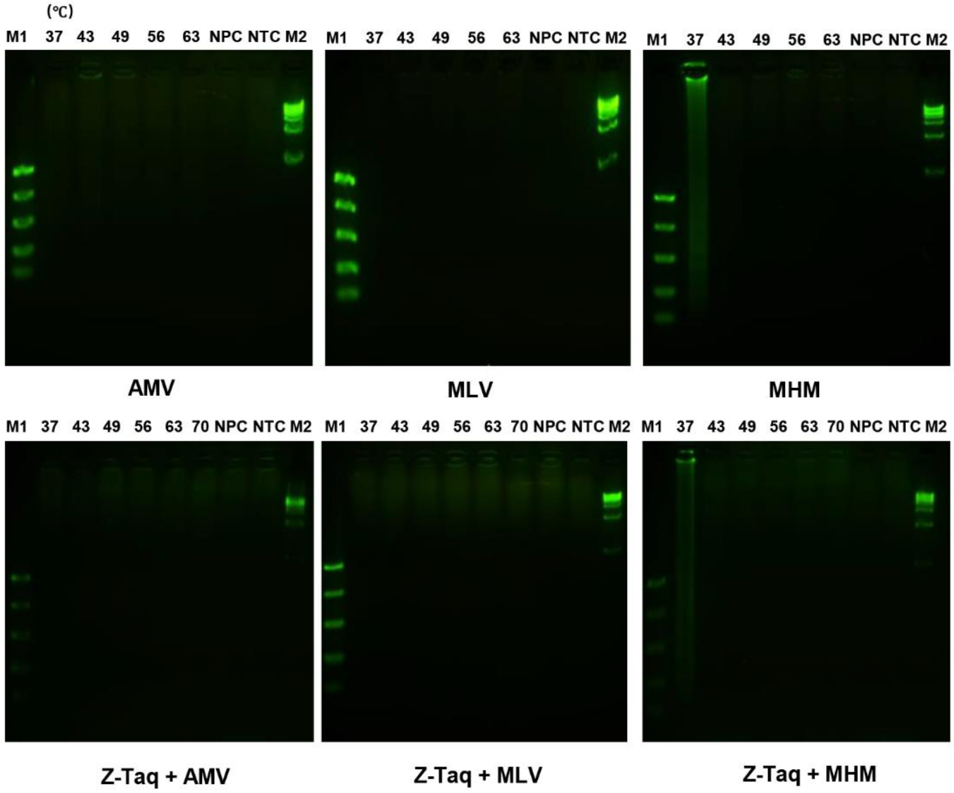

Figure S8. Verification of UIMA by mixture of DNA polymerase without reverse transcriptase (RT) activity and reverse transcriptase. Experiment with combining DNA polymerase without RT activity (z-Taq) and RT such as AMV, MLV, and MHM, respectively. The reactions were performed at different temperatures for 180 min and the products were analyzed by 2.5% agarose gel. For AMV and MLV, NTC and NPC were performed at 43ºC; For MHM, NTC and NPC were performed at 37ºC. The same set was for tests mixture polymerases. NTC: no-template control; NPC: no-primer control. The groping of gels cropped from different gels. Exposure time is 5 s.

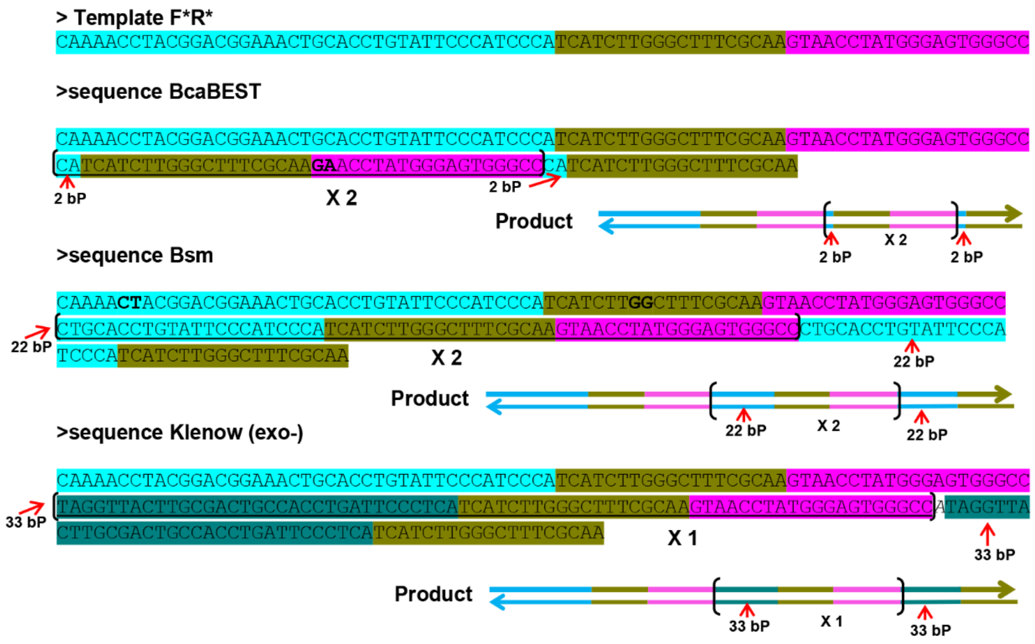

Figure S9. Sequence analysis of UIA products by different DNA polymerase. The products of reactions amplificated for 180 min were cloned into the T-vector and sequenced. The braces showed the position of the repeating units. The numbers under the underlines showed the number of consecutive repeats in the sequence. The italic base in the sequence indicated additional base. Horizontal arrows denoted the 5'-3' direction of sequences.

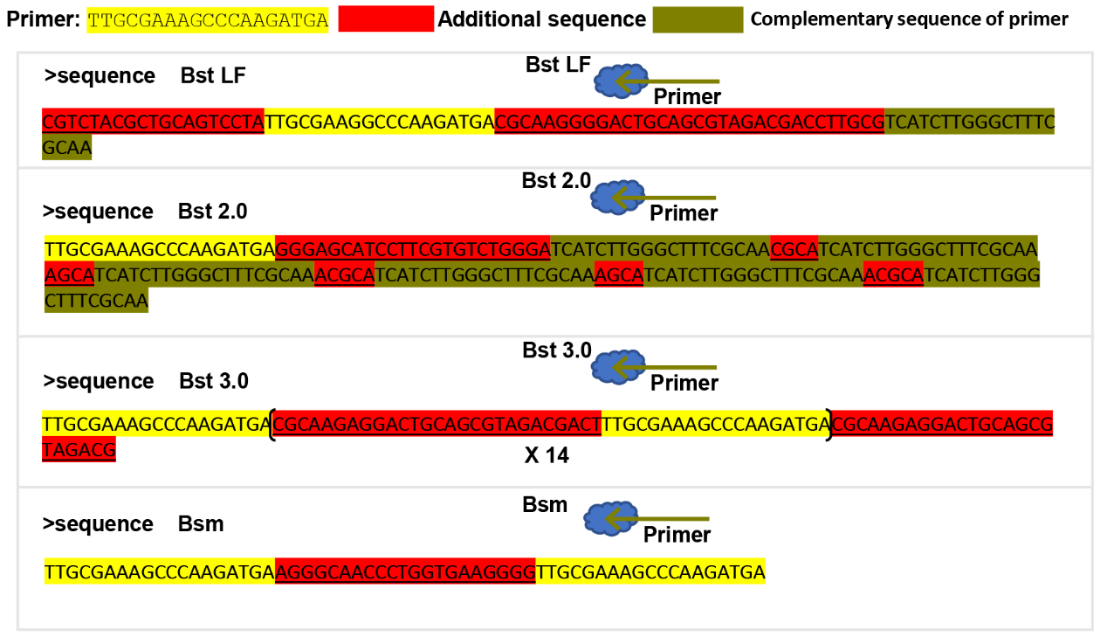

Figure S10. Sequence analysis of NTCs by different DNA polymerases. The products of reactions amplificated for 180 min were cloned into the T-vector and sequenced. The braces showed the position of the repeating units. The numbers under the underlines showed the number of consecutive repeats in the sequence. The highlight of red means the additional sequence. Others mean the primer or complementary sequence of primer. Horizontal arrows denoted the 5'-3' direction of sequences.

>Template HBV:

CTGCTCAAGGAACCTCTATGTTTCCCTCATGTTGCTGTACAAAACCTACGGACGGAAACTGCACCTGTATTCCCATCC
CATCATCTTGGGCTTTCGCAA

Primer HBV-RL: AGTTTCCGTCCGTAGGTTTTG

>Sequence result

12 bP

AGTTTCCGTCCGTAGGTTTTGTACGGATGGAAACTGCACCTGTATTCCCATCCCATCATCTTGGGCTTTCGCAACATG
TTGCTGTACAAAACCTACGGACGGAAACTGCACCTGTATTCCCATCCCATCATCTTGGGCTTTCGCAACATGTTGCTG
TACAAAACCTACGGACGGAAACT

12 bP

X 4

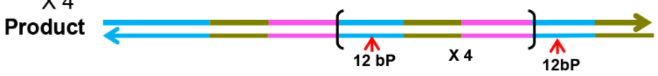

> Template HPV-18

GAAATAGATGGAGTTAATCATCAACATTTACCAGCCCGACGAGCCGAACCACAACGTCACACAATGTTGTGTATGTGT
TGTAAGTGTGAAGCCAGAATTGAGCTAGTAG

Primer HPV-18-RL: CTTCACACTTACAACACATACACA

>Sequencing result

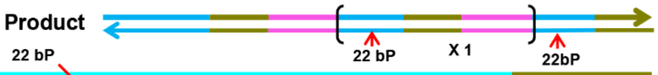

22 bP

GAAATAGATGGAGTTAATCATCAACATTTACCAGCCCGACGAGCCGAACCACAACGTCACACAATGTTGTGTATGTGT
TGTAAGTGTGAAGCCAGAATTGAGCTAGTAGAGCCGAACCAC***AC***ACACAATGTTGTGTATGTGTTGTAAGTGTGAAGC
CAGAATTGAGCTAGTAGAGCCGAACCAC***AC***ACACAATGTTGTGTATGTGTTGTAAGTGTGAAG X 1

22 bP

Figure S11. Sequence analysis of universality assay's products. The products of reactions amplificated at 63 ℃ for 180 min were cloned into the T-vector and sequenced. The braces showed the position of the repeating units. There was a mispairing in the 5' -end of HBV. The numbers under the underlines showed the number of consecutive repeats in the sequence. The bold and italic bases indicated deletion mutations. Horizontal arrows denoted the 5'-3' direction of sequences.

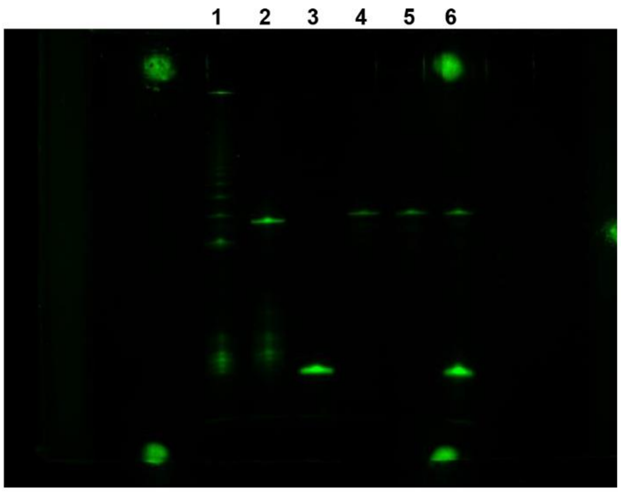

Figure S12. Full-length gels of Figure 6B.
